# Epileptogenesis in neurocutaneous disorders with focus in Sturge Weber syndrome

**DOI:** 10.12688/f1000research.7605.1

**Published:** 2016-03-18

**Authors:** Anna Pinto, Mustafa Sahin, Phillip L. Pearl

**Affiliations:** 1 Department of Neurology, Boston Children's Hospital, Boston, Massachusetts, USA; 2 Division of Epilepsy, Department of Neurology, Boston Children's Hospital, Boston, Massachusetts, USA; 3 Department of Neurology, Dartmouth Hitchcock, Manchester, New Hampshire, USA

**Keywords:** epilepsy, Sturge Weber syndrome, seizures, epileptogenesis, neurocutaneous syndromes

## Abstract

Epilepsy is a major morbidity in Sturge Weber syndrome, a segmental vascular neurocutaneous disorder classically associated with facial angiomas, glaucoma, and leptomeningeal capillary-venous type vascular malformations. The extent of the latter correlates with neurological outcome. Post-zygotic mosaicism for the activating mutation p.R183Q of the *GNAQ* gene has been identified as the major cause. *GNAQ* encodes for an alpha subunit of a heterotrimeric G protein critical to blood vessel development. The earlier the timing of the mutation in development, the more severe the involvement, e.g. from isolated port-wine stains to the full syndrome. The strongest predictors of adverse outcomes are MRI and the presence of angiomas involving any part of the forehead, delineated inferiorly from the outer canthus of the eye to the top of the ear, and including the upper eyelid.  The neurological course may be progressive and the typical constellation of symptoms is focal onset seizures, hemiparesis, headache, stroke-like episodes, behavior problems, intellectual disability, and visual field deficits. Antiseizure medications are effective in about half of patients. The presence of localized seizures, focal neurological deficits, and drug resistant epilepsy indicate epilepsy surgical evaluation. Earlier seizure onset, i.e. before six months of age, is associated with a more severe course with significant residual deficits. Factors contributing to epileptogenesis include decreased brain tissue perfusion due to abnormal venous drainage, anoxic injury contributing to cerebral calcification, breakdown of the blood-brain barrier, and the presence of developmental cortical malformations. Pre-symptomatic prophylactic treatment may be a future option to modify the course of the disease including the associated epileptogenesis.

## Introduction

The phakomatoses or neurocutaneous syndromes comprise a heterogeneous group of diseases that are mostly hereditary and characterized by the association of skin lesions with a variety of central and/or peripheral nervous system manifestations. Of the common phakomatoses, tuberous sclerosis and Sturge-Weber syndrome (SWS) feature epilepsy as a major morbidity to a complex phenotype of variable severity. SWS is a segmental vascular neurocutaneous disorder classically associated with facial angiomas known as port-wine stains, glaucoma associated with vascular ocular abnormalities, and leptomeningeal capillary-venous type vascular malformations. The extent of the latter correlates with the neurological outcome.

Recent groundbreaking research identified a somatic activating mutation of the gene *GNAQ* as the likely cause of the majority of cases of SWS as well as non-syndromic port-wine stains. Interestingly, the timing of the somatic mutation in *GNAQ* during development likely impacts the clinical phenotype ^1^.

The neurological manifestations in SWS are often progressive. Brain involvement is common with the capillary malformation causing progressive epilepsy and cerebral atrophy. The extent of the capillary malformation is correlated with the severity of seizures, extent of atrophy, and cognitive outcome. The pathophysiological processes leading to epileptogenesis and atrophy are not entirely known. This review outlines possible mechanisms of epileptogenesis in SWS ^2^.

## Pathophysiology

A somatic activating mutation in the *GNAQ* (p.R183Q) gene was identified in the affected skin of individuals with non-syndromic port-wine stains and in SWS patients ^1^. Thus, post-zygotic mosaicism for this *GNAQ* mutation has been described as the major cause of SWS.

*GNAQ* encodes Gαq, an alpha subunit of the heterotrimeric G-protein that links G-protein-coupled receptors to activation of phospholipase C (PLC), transient increases in cytosolic calcium, and activation of Rac and Rho. The arginine (R) residue at position 183 in Gαq is a conserved amino acid in the GTP-binding pocket. R183Q mutation leads to a decrease in function of the GTPase and to constitutive activation of downstream effector pathways. Several of the G-protein-coupled receptors linked to Gαq, such as Gβ and Gγ subunits, are critical to blood vessel development and function; therefore, the abnormal signaling may result in vascular malformations ^3^.

**Figure 1.**
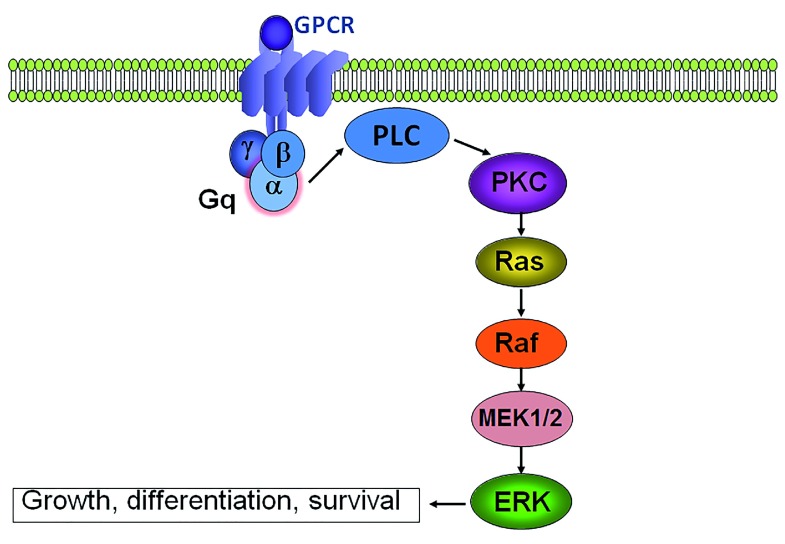
Schematic representation of *GNAQ* (encodes for Gαq) signaling via RAS/ERK pathway.

Gαq effectors increase downstream signaling through the RAS signaling pathway ( Figure 1), and this is an implicated mechanism to explain the increased cell proliferation and inhibition of apoptosis in the affected skin and leptomeningeal capillary malformation samples in patients with SWS. The cell of origin affected by the mutation is not yet known ^4, 5^. Recent research showed that endothelial cells in capillary malformations are enriched for *GNAQ* mutations and are likely responsible for the pathophysiology underlying capillary malformations ^6^. It is likely that the mutation occurs earlier in development in SWS than in isolated port-wine stains, thus affecting an earlier progenitor with wider potential downstream effects. Somatic mutations in *GNAQ* at other amino acids are also seen in uveal melanoma and more recently in the extended spectrum of clinical presentation from phakomatosis pigmentovascularis (PPV) to extensive dermal melanocytosis ^7^. Based on the diversity of conditions and spectrum of severity, it seems that the mutation occurring at different times in development will influence the phenotype and severity of the condition. Microscopic examination of SWS brain tissue shows deposition of calcium in the cortex, hypoplastic blood vessels, gliosis, and sometimes loss of neurons or focal cortical dysgenesis ^8, 9^. The current evidence suggests that observed malformation of brain development in patients with SWS is likely secondary to abnormal vascular development concomitant to the cortical developmental stages.

## Clinical considerations on neurological aspects of SWS

The clinical course of SWS is variable and can be devastating. In a cohort of 192 patients with facial port-wine stains, a strong predictor of adverse outcomes was an angioma involving any part of the forehead, delineated at its inferior border by a line joining the outer canthus of the eye to the top of the ear, and including the upper eyelid ^10^. Abnormal MRI was a better predictor of all clinical adverse outcome measures than port-wine stain distribution, although for practical reasons guidelines based on clinical phenotype are proposed. Typically, the progressive neurological problems evolve over time causing medication-resistant epilepsy and brain atrophy. The constellation of symptoms is characterized by focal onset of seizures, hemiparesis, headache, stroke-like episodes, behavioral problems, intellectual disability, and visual field defects.

Seizures occur in 75–80% of all SWS patients and in over 90% of patients with bilateral involvement. Onset of seizures occurs in 75% of children before 1 year of age, 86% by 2 years of age, and 95% by the age of 5 years. Medications are effective in preventing seizures in approximately 50% of patients. Patients with localized seizures, focal neurological deficits, and drug-resistant epilepsy should be considered for epilepsy surgery ^11, 12^.

Based on the revised International League Against Epilepsy (ILAE) classification, the most common seizure type is focal seizures with an observable motor component and without impairment of awareness. Seizures with variable degrees of impairment of consciousness are also frequently observed along with autonomic features and evolution to bilateral convulsive events. Seizures can be subtle, and prompt recognition of an epileptic episode is important because prolonged seizures and status epilepticus are commonly seen ^13^.

About 30% of cases may have onset of seizures during febrile episodes, and there is an increased susceptibility for fever-induced seizures at any age ^14^. Apneic seizures have been associated with SWS and present in nearly half of drug-resistant patients who require epilepsy surgery (Pinto *et al.*, in press). Unusual seizure types such as ictal laughter have also been described in SWS patients ^15^. Children with epilepsy due to focal lesions can develop secondary bilateral synchrony. There are also reports of myoclonic astatic seizures, infantile spasms including asymmetric features, and prolonged post-ictal paralysis ^15, 16^.

Some patients with SWS may present with initial clusters of frequent epileptic events and then remain seizure-free for several months or years. Remissions may last several months before recurrence occurs. The variable course of the epilepsy renders surgical disposition difficult, and the trajectory of associated developmental progress or decline is often a deciding factor ^14^.

A descriptive study of 77 children and adults with epilepsy secondary to SWS disclosed a dichotomy based on age at seizure onset. Early onset patients (onset of seizures before 6 months of age) had a severe early course with significant residual deficits, while late-onset patients (onset of seizures after 6 months of age) did better. Focal cerebral atrophy worsened in early onset cases. The course of the epilepsy in the late-onset cohort stabilized after 5 years of age in most cases. The authors described that aspirin use correlated with stable course of epilepsy in six patients. Leptomeningeal enhancement appears to increase on imaging studies during acute events before returning to baseline, suggesting that extent of disease is probably best judged during the interictal state. The use of aspirin routinely is still controversial ^17, 18^.

## Electroencephalography in SWS

The typical electroencephalogram (EEG) in patients with SWS consists of asymmetric background frequencies and voltages ^19^. A large cohort study with emphasis on EEG evolution analyzed 81 EEGs from 44 children and adults with SWS. This study documented the evolutional changes that have been previously described. Recordings evolve to show asymmetric slowing over the course of 1 year; 2 to 3 years later, interictal epileptiform activity with focal sharp waves and increasingly frequent spike discharges appear. One limitation of the study was the potential modification of the EEG pattern due to modern anti-seizure medications or treatment with low-dose aspirin. The detailed EEG analysis did not, however, show a correlation between the EEG severity score and clinical function or seizure control ^20^.

Atypical findings in ictal EEG have been reported. For instance, patients with unilateral brain insults have shown ictal contralateral slow waves. A patient reported with this pattern became seizure free after hemispherectomy, and the abnormalities seen in the healthy contralateral hemisphere were not a sign of an ictal hemisphere but instead could indicate prominent ischemic changes resulting from a vascular steal phenomenon during the seizure ^21^.

Most recently, analysis of infraslow EEG activity (ISA) was effective in identifying refractory subclinical focal status epilepticus in a pediatric patient who presented with a 96-hour refractory encephalopathy and non-ischemic hemiparesis, which successfully resolved after midazolam administration. In general, ISA has shown potential in the evaluation of patients with epilepsy and in the differentiation between focal and generalized epilepsies ^22^.

## Neuroimaging in SWS

Contrast-enhanced MR studies are the most accurate single imaging studies to demonstrate the extent of brain involvement; contrast enhanced T2-weighted FLAIR images improve detection of leptomeningeal disease compared to post-contrast T1-weighted images ^22– 24^. Other typical features include enlargement of the choroid plexus, cortical calcifications, and cerebral atrophy. The demonstration of the extent of the capillary malformation is critical in determining the patient’s prognosis and the necessary approach for cortical resection for epilepsy surgery ^24, 25^. Dynamic MR perfusion studies suggest hypoperfusion in the underlying brain is connected to the abnormal pial angioma. Cerebral hypoperfusion is essentially secondary to impaired venous drainage. Perfusion imaging reveals increased mean transit time and in more severe cases reduced regional blood flow ^25– 27^. Proton MR spectroscopy of the affected brain region reveals elevated choline, reduced N-acetylaspartate, and slightly elevated lactate, probably resulting from ongoing ischemia and secondary gray and white matter injury. The increased choline peak is possibly related to accelerated myelination observed in early stages of SWS. Multimodality neuroimaging can be of use to identify areas at risk for future metabolic and functional deterioration ^24^.

## Positron emission tomography/single photon emission computed tomography

Patients with refractory seizures, including those with SWS, often undergo functional studies in preparation for surgery. Given the unique neurovascular coupling mechanism in SWS, functional studies should be interpreted carefully because the vascular malformation in SWS is associated with impairment of the cerebral hemodynamic response to seizure activity.

Increased glucose metabolism detected by positron emission tomography (PET) has been observed in the lesioned hemisphere. A prospective cohort study was designed with the objective of verifying the significance of interictal hypermetabolism on PET images of children with SWS ^28^. The authors studied the prevalence and clinical correlates of focal increases in cerebral glucose metabolism with seizure onset as well as evolution to drug-resistant epilepsy. Patients with foci of increased cortical glucose metabolism were significantly younger than those with decreased cortical metabolism. Interictal glucose hypermetabolism in young children with SWS is most often seen within a short time before or after the onset of first clinical seizures, and this finding was associated with the presumed period of epileptogenesis. Increased glucose metabolism detected by PET may predict future demise of the affected cortex based on a progressive loss of metabolism and may be an imaging marker of the most malignant cases of intractable epilepsy requiring surgery in SWS ^28^.

Single photon emission computed tomography (SPECT) detects cerebral blood flow (CBF) asymmetry in infants with SWS, which tends to shift with age. The cortex involved in the vascular malformation is hyperperfused during the first year of life before seizure onset. The classic hypoperfusion appears after 1 year of age, even in patients who do not experience seizures ^29^. The hypoperfusion seems to result from post-ictal phenomena as well as chronic ischemia. SPECT imaging generally demonstrates hypoperfusion in the diseased tissue. The affected cerebral tissue typically shows increased CBF during the ictal state. Decreased blood flow during seizures, however, can also be observed. These findings point to the variable results of functional studies in SWS that might lead to miscalculation of the lesioned area while planning for surgery ^30^.

Previous studies using SPECT imaging have shown massive steal phenomena in affected areas during seizures, which could lead to a critical ischemic condition in remote brain regions. Therefore, progressive worsening could be partially explained by repeated seizures with severe ischemia of previously unaffected brain parenchyma ^21^.

## Role of ischemia in epileptogenesis

The hallmark signs of SWS are tortuous and abnormal vascular structures in thickened leptomeninges. These vessels all have similar appearance, caliber, and morphological characteristics. The areas of capillary malformation are described as capillary-venous type vascular malformations. Underlying brain tissue can be atrophic and display neuronal loss, astrogliosis, dysgenic cortex, and calcification in the cortical layers. The cortical vessels underlying the meningeal angiomas are thin-walled, narrowed by hyalinization and subendothelial proliferation, and increased in number. Cerebral angiography demonstrates an aberrant pattern of both the arterial and venous cerebral circulation. Along with areas of arterial thrombosis, there is abnormal venous drainage, with manifest paucity of the superficial draining veins, venous occlusions, and alternative venous flow through deep subependymal channels ^11, 12^.

Low brain tissue perfusion due to abnormal venous drainage is part of the central mechanism of brain damage in SWS. Decreased quantitative white matter perfusion has been associated with frequent seizures, duration of epilepsy, and brain atrophy ^31^. The authors used MR perfusion-weighted imaging (PWI) of the affected cerebral white matter to study dynamic perfusion abnormalities in children with SWS. They concluded that increased perfusion in mostly younger patients may represent a transient phenomenon before severe brain atrophy occurs in the affected brain regions. Decreased perfusion is associated with high seizure frequency, long epilepsy duration, and severe brain atrophy, suggesting a detrimental effect of chronic seizures on brain structure and function.

Regional perfusion and cortical metabolic abnormalities can extend beyond lobes affected by leptomeningeal vascular malformations and are related to epilepsy in SWS. PWI along with fluorodeoxyglucose PET (FDG-PET) has been used to study the relationship between regional metabolic and perfusion abnormalities in SWS. While decreased perfusion was associated with hypometabolism in most cases, increased regional CBF was commonly associated with relatively mild or no hypometabolism. Despite a general correlation between perfusion and metabolism, increased white matter perfusion with preserved cortical metabolism in overlying cortex is a common pattern of a perfusion/metabolic mismatch. This may represent a disease stage where cortical function is preserved while increased white matter perfusion provides collateral drainage of cortex via the deep venous system ^32^.

## Calcification as source of seizure

Anoxic injury to endothelial cells secondary to impaired venous drainage may contribute to cerebral calcification. However, enhancement of leptomeningeal vessels and enlarged deep venous vessels suggest breakdown of the blood–brain barrier; thus, calcification could also result from increased blood vessel permeability. The calcification typically occurs exclusively in areas subjacent to the abnormal vasculature. It begins in the subcortical white matter and later develops in the cortex affecting predominantly layers II and III. The common sites are the parietal-occipital regions ^33^.

In a study of 15 children with unilateral SWS, the degree of cortical calcification was assessed using susceptibility-weighted imaging (SWI) while perfusion status was quantified using dynamic susceptibility contrast PWI (DSC-PWI). Prospective data show that prominent calcification in the affected hemisphere reflects markedly decreased perfusion in underlying white matter and is associated with more severe epilepsy in SWS patients ^33^.

## Epilepsy in SWS associated with cortical malformation

Although some malformations of cortical development are caused by environmental insults that occur during cortical development *in utero*, genetic factors also play a critical role in the pathogenesis of many cortical malformations ^34^. Morphological anomalies have been reported on histological examination of surgical samples from SWS patients. One of the common findings associated with developmental cortical malformations in patients with SWS is polymicrogyria. Like other types of cortical malformations, there is a range of severity from very focal, unilateral forms to extensive bilateral involvement. Traditionally, many cases of polymicrogyria were thought to result from environmental insults during later stages of cortical development, typically during cortical organization following neuronal migration. However, some genetic causes have recently been identified ^9, 35^.

## Future research

Given the high incidence of epilepsy in patients with SWS, pre-symptomatic prophylactic treatment has been proposed. A prospective study showed possible improvement in cognitive impairment in a group that were given prophylactic treatment ^36^. Perhaps the use of anti-seizure drug therapy before clinical seizure onset can modify the course of severe epilepsy in SWS patients. Multicenter studies with appropriate patient selection and medication choices will be helpful to study this possibility.

SWS is a progressive and potentially devastating neurocutaneous disorder that typically evolves over time and may result in drug-resistant epilepsy and brain atrophy. Future research aimed at understanding the mechanisms of this condition and comorbidities will have a high impact on our understanding of early stages of brain involvement and will provide the means to assess responses to novel interventions. Multicenter studies are imperative to determine possible candidates for early biomarkers for brain involvement prior to irreversible structural changes.
